# Norovirus GII.2[P16] strain in Shenzhen, China: a retrospective study

**DOI:** 10.1186/s12879-021-06746-9

**Published:** 2021-10-30

**Authors:** Jing Wang, Miao Jin, Hailong Zhang, Yanan Zhu, Hong Yang, Xiangjie Yao, Long Chen, Jun Meng, Guifang Hu, Yaqing He, Zhaojun Duan

**Affiliations:** 1 Wuhan Wuchang Hospital, Wuchang Hospital Affiliated to Wuhan University of Science and Technology, Wuhan, 430063 China; 2 NHC Key Laboratory of Medical Virology and Viral Diseases, National Institute for Viral Disease Control and Prevention, Chinese Center Control and Prevention, Beijing, 102206 China; 3 Shenzhen Center for Disease Control and Prevention, Shenzhen, 518055 China; 4 Department of Epidemiology, School of Public Health, Southern Medical University, Guangzhou, China

**Keywords:** Norovirus, Epidemiology, GII.2[P16] strain, Recombinant, Evolution

## Abstract

**Background:**

Norovirus (NoV) is the main cause of non-bacterial acute gastroenteritis (AGE) outbreaks worldwide. From September 2015 through August 2018, 203 NoV outbreaks involving 2500 cases were reported to the Shenzhen Center for Disease Control and Prevention.

**Methods:**

Faecal specimens for 203 outbreaks were collected and epidemiological data were obtained through the AGE outbreak surveillance system in Shenzhen. Genotypes were determined by sequencing analysis. To gain a better understanding of the evolutionary characteristics of NoV in Shenzhen, molecular evolution and mutations were evaluated based on time-scale evolutionary phylogeny and amino acid mutations.

**Results:**

A total of nine districts reported NoV outbreaks and the reported NoV outbreaks peaked from November to March. Among the 203 NoV outbreaks, 150 were sequenced successfully. Most of these outbreaks were associated with the NoV GII.2[P16] strain (45.3%, 92/203) and occurred in school settings (91.6%, 186/203). The evolutionary rates of the RdRp region and the VP1 sequence were 2.1 × 10^–3^ (95% HPD interval, 1.7 × 10^–3^–2.5 × 10^–3^) substitutions/site/year and 2.7 × 10^–3^ (95% HPD interval, 2.4 × 10^–3^–3.1 × 10^–3^) substitutions/site/year, respectively. The common ancestors of the GII.2[P16] strain from Shenzhen and GII.4 Sydney 2012[P16] diverged from 2011 to 2012. The common ancestors of the GII.2[P16] strain from Shenzhen and previous GII.2[P16] (2010–2012) diverged from 2003 to 2004. The results of amino acid mutations showed 6 amino acid substitutions (*77E, R750K, P845Q, H1310Y, K1546Q, T1549A) were found only in GII.4 Sydney 2012[P16] and the GII.2[P16] recombinant strain.

**Conclusions:**

This study illustrates the molecular epidemiological patterns in Shenzhen, China, from September 2015 to August 2018 and provides evidence that the epidemic trend of GII.2[P16] recombinant strain had weakened and the non-structural proteins of the recombinant strain might have played a more significant role than VP1.

**Supplementary Information:**

The online version contains supplementary material available at 10.1186/s12879-021-06746-9.

## Background

Norovirus (NoV), which is the main cause of non-bacterial acute gastroenteritis (AGE) worldwide, can infect all age groups, especially children under 5 years of age. According to estimations, NoV is annually associated with 900,000 clinic visits amongst children in industrialized countries and up to 200,000 deaths of children in developing countries [1, 2]. In general, NoV circulates in colder weather and causes gastrointestinal symptoms such as vomiting, diarrhoea and abdominal pain. NoV outbreaks are frequently reported in semi-closed institutions, such as hospitals, nursing homes, schools, and childcare centres [3].

NoV belongs to the *Caliciviridae* family and can be divided into 10 genogroups (GI ~ GX), of which GI, GII and GIV infect humans. GI and GII are responsible for the majority of human diseases and can be further divided into nine (GI.1–GI.9) and 27 (GII.1–GII.27) genotypes based on the diversity of VP1 [4]. The full-length single stranded RNA genome is approximately 7.5 ~ 7.7 kb, with three open reading frames (ORFs) [5]. The first 5 kb closest to the 5’ end of the genome is ORF1, which encodes non-structural proteins, including N-terminal protein (P48), NTPase, 3A protein (P22), VPg (viral genomic junction protein), 3 C-like protein (Pro) and RNA-dependent RNA polymerase (RdRp) [6]. These proteins are important for the replication of NoV. ORF2 is 1.6 kb in length and encodes the major structural protein VP1, which constitutes the main capsid structure and is responsible for the infectivity and antigenicity of NoV [7]. VP1 contains a well-conserved shell (S) domain and a protruding (P) domain, and the latter is divided into two subdomains, P1 and P2 [8]. Furthermore, the P2 region is considered a hypervariable part of the genome because the domain encodes the receptor-binding domain, which is responsible for histoblood group antigen (HBGA) binding, and important epitopes targeted by antibodies that inhibit binding [9, 10]. ORF3 is 0.6 kb and encodes the minor structural protein (VP2) [11].

The global dominant epidemic variant strain is generally GII.4. Since 2002, new GII.4 variants have emerged every 2–3 years and replaced the previously predominant GII.4 strains, resulting in epidemics and sometimes global pandemics of AGE including GII.4 Hunter2004, GII.4 Yerseke2006a, GII.4 Den Haag2006b, GII.4 New Orleans2009, GII.4 Sydney2012 [12]. However, during the winter of 2014–2015, a novel GII.17 strain initially emerged in Guangdong Province, surpassing GII.4-caused NoV infections [13]. Moreover, in late 2016, a novel GII.2[P16] recombinant strain in which the RdRp region clustered closely with GII.3[P16]/GII.4 Sydney2012[P16] strains (2015–2017) and the VP1 sequence clustered closely with GII.2[P16] strains (2011–2012), leaded to rapidly increasing AGE outbreaks in China [14] and during a short time, the GII.2[P16] recombinant strain swept through Japan, Italy, Germany [15–17]. The first GII.2[P16]-positive sample was also detected in Guangdong Province [14].

Shenzhen is one of the most important cities in Guangdong Province. However, information about NoV outbreaks in this region is limited. This retrospective study aimed to determine the genotypic diversity of NoV strains in outbreaks and the genetic characteristics of the GII.2[P16] strain in Shenzhen, China, from September 2015 to August 2018.

## Methods

### The surveillance of NoV outbreaks

Faecal specimens in AGE outbreaks submitted to the Shenzhen Center for Disease Control and Prevention (Shenzhen CDC) by District Centers for Disease Control and Prevention (district-level CDCs) from September 2015 to August 2018 were obtained. District-level CDCs are responsible for conducting outbreak investigations, including providing epidemiological and clinical information. The Shenzhen CDC performs NoV detection and genotyping on the specimens. The NoV outbreaks were identified as > 5 acute gastroenteritis cases within 3 days after exposure in a common setting where > 2 samples (whole faecal, rectal swab, or vomitus) had been laboratory confirmed as NoV.

### Detection of NoV by real-time RT-PCR

For faecal specimen analysis, a 10% suspension was prepared by mixing 0.1 g stool with 1 mL phosphate-buffered saline (pH 7.2). Viral RNA was extracted from the clarified stool suspension using the Viral Nucleic Acid Extraction Kit II (Geneaid, China), after which the viral RNA was examined by real-time reverse transcription polymerase chain reaction (real-time RT-PCR) using Ag-Path Kit (Applied Biosystems, USA) with primers (Cog1F, Cog1R, Cog2F, and Cog2R) and TaqMan probe (Ring 1E and Ring 2) (Additional file 1: Table S1). The cycling conditions were described previously [18]. A negative control containing DEPC water and 2 positive controls containing RNA of NoV GI and GII were included in each experiment. Samples were scored as positive if the cycle threshold values were ≤ 40 and the positive and negative controls showed the expected values.

### Genotyping of NoV by conventional RT-PCR

NoV-positive samples were then amplified by conventional reverse transcription and PCR (RT-PCR) using a one-step RT-PCR Kit (QIAGEN, Germany). Before October 2016, the primer sets G1SKF/G1SKR and COG2F/G2SKR were used for VP1 genotyping to detect GI (330 bp) and GII (387 bp), respectively [19] (Additional file 1: Table S1). After October 2016, the primer sets MON432/G1SKR and MON431/G2SKR were used to amplify both the partial RdRp region and VP1 sequence of GI (543 bp) or GII (557 bp), respectively [20] (Additional file 1: Table S1).

### Genotyping analysis

Genotypes were confirmed by BLAST and an automated online NoV genotyping tool offered by the Netherlands National Institute for Public Health and the Environment (RIVM, http://www.rivm.nl/mpf/norovirus/typingtool) [21].

### Genome amplification of strains GII.2[P16] of NoV

All the samples of the genomes of the strains genotyped as GII.2[P16] were further amplified. cDNA was obtained by reverse transcription of viral RNA using a SuperScript III kit (Invitrogen, USA). Six primer sets (Additional file 1: Table S1) were designed based on the whole genome of GII.2[P16] reference strain (GenBank accession No. KY421121). Genome amplification was carried out using touchdown PCR with a PrimeSTAR ® Max DNA polymerase kit (Takara, Japan). The samples contained 10 µl PrimeSTAR buffer, 7 µl DEPC water, 1 µl forward primer (20 M), 1 µl reverse primer (20 M) described in Additional file 1: Table S1, and 1 µl cDNA preparation in a total volume of 20 µl. The samples were initially heated at 95 °C for 1 min, followed by 10 cycles consisting of 98 °C for 10 s, 60 °C (decreasing incrementally by 0.5 °C per cycle, 1F/1R, 3F/3R,4F/4R and 6F/VN3T20 chose 60 °C, 3F/3R and 5F/5R chose 62 °C) for 30 s, and 72 °C for 2 min 30 s, then 95 °C for 1 min, followed by 32 cycles at 98 °C for 10 s, 55 °C (1F/1R, 3F/3R,4F/4R and 6F/VN3T20 chose 55 °C, 3F/3R and 5F/5R chose 57 °C) for 30 s, and 72 °C for 2 min 30 s, culminating with a final cycle at 72 °C for 2 min 30 s. A negative control (PCR water) and positive-control samples were included in all experiments. The PCR products were analysed by electrophoresis on 1.5% (wt/vol) agarose gels containing 1 × Tris–acetate-EDTA buffer. The molecular size markers were run in parallel on all gels.

### Phylogenetic analysis of the RdRp region and VP1

To evaluate the evolution of the NoV GII.2[P16] strain in Shenzhen, the full-length RdRp region or VP1 sequence from this study and all the sequences of the full-length RdRp region or VP1 sequence we found in GenBank as of September 2016 were collected. Phylogenetic trees were constructed using the Markov chain Monte Carlo (MCMC) method with the strict molecular clock in BEAST software v 1.8.2. The best substitution models were TN93 (Tamura-Nei) + G (Gamma) and TN93 (Tamura-Nei) + G (Gamma) + I (Invariable) for the RdRp region and VP1 sequence, selected by MEGA 6.0 using the BIC method [22]. MCMC chains were run for 1.0 × 10^8^ steps for the RdRp region sequences and 2.0 × 10^8^ steps for the VP1 sequences. Effective sample sizes greater than 200 were confirmed by the Tracer.The final result was visualized using the FigTree software v1.4.3.

### Amino acid mutations of the non-structural region and VP1

To evaluate the impact of the intergenic recombination of the non-structural region and VP1, the amino acid mutations of the non-structural region and VP1 among different genotypes were analysed by MEGA 6.0.

### Statistical analysis

The difference between GII.2 NoV detection rates in the age distribution were compared using Fisher's exact test by SPSS Statistics software v.22.0 through dominant school settings (childcare centre, primary school, middle school), and a *p*-value less than 0.05 was considered statistically significant.

### Nucleotide sequence accession numbers

The datasets generated during the current study for the GII.2[P16] strain sequences are available in the GenBank and the accession numbers are MK729081, MK681452, MK614124-MK614161, MK720506-MK720583, and MK692738-MK692654.

## Results

### NoV outbreak settings and geographical locations

**Fig. 1:**
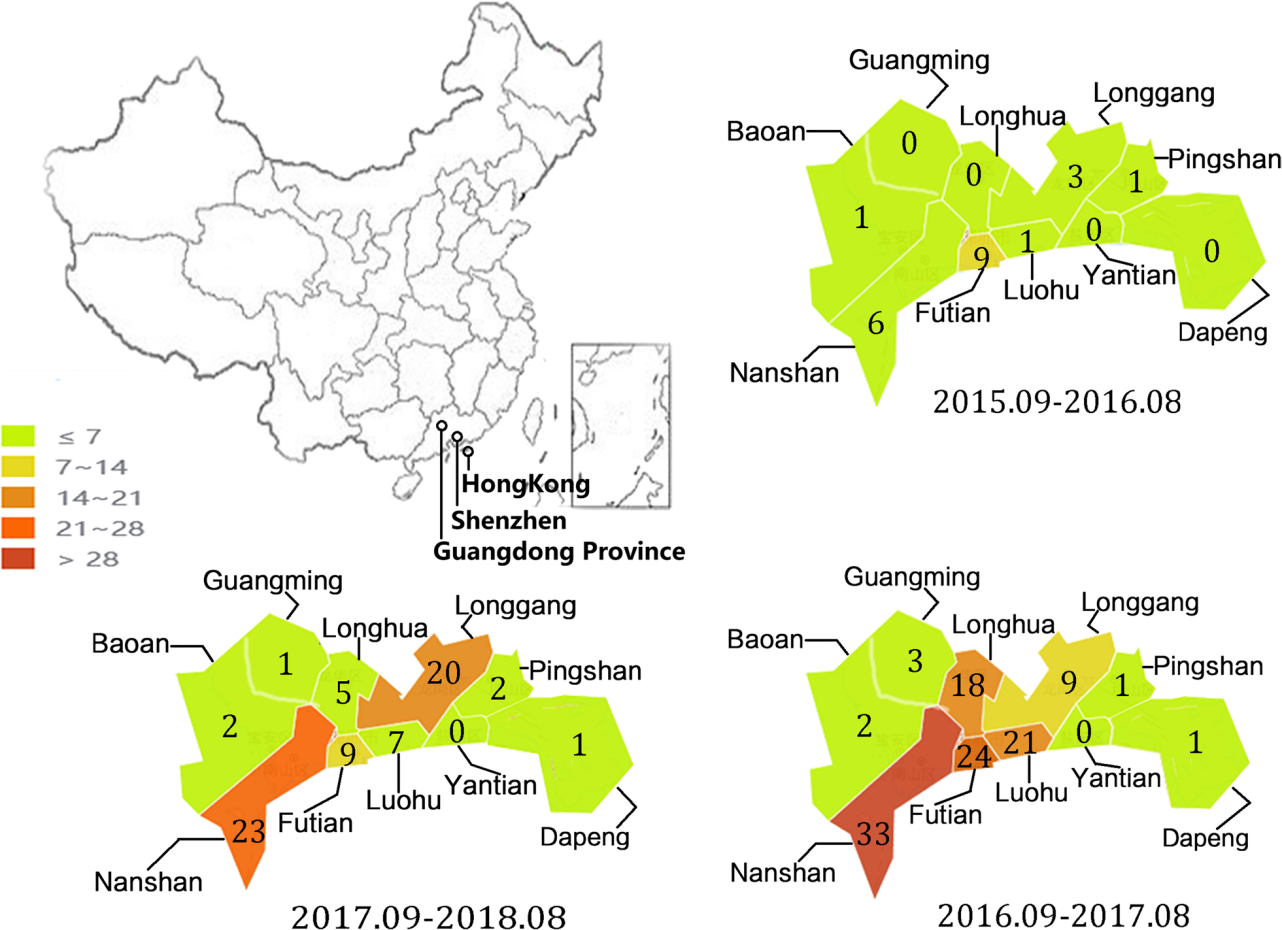
Geographical distribution of NoV outbreaks in Shenzhen from September 2015 to August 2018. A map of China is shown to localize the studied city, and the numbers on the map denote the numbers of outbreaks in the region. The different colours represent different numbers of outbreaks, which are marked on the left. The names of the counties are also shown. The map was created by an online tool offered by Dituhui (http://c.dituhui.com/apps)

**Fig. 2:**
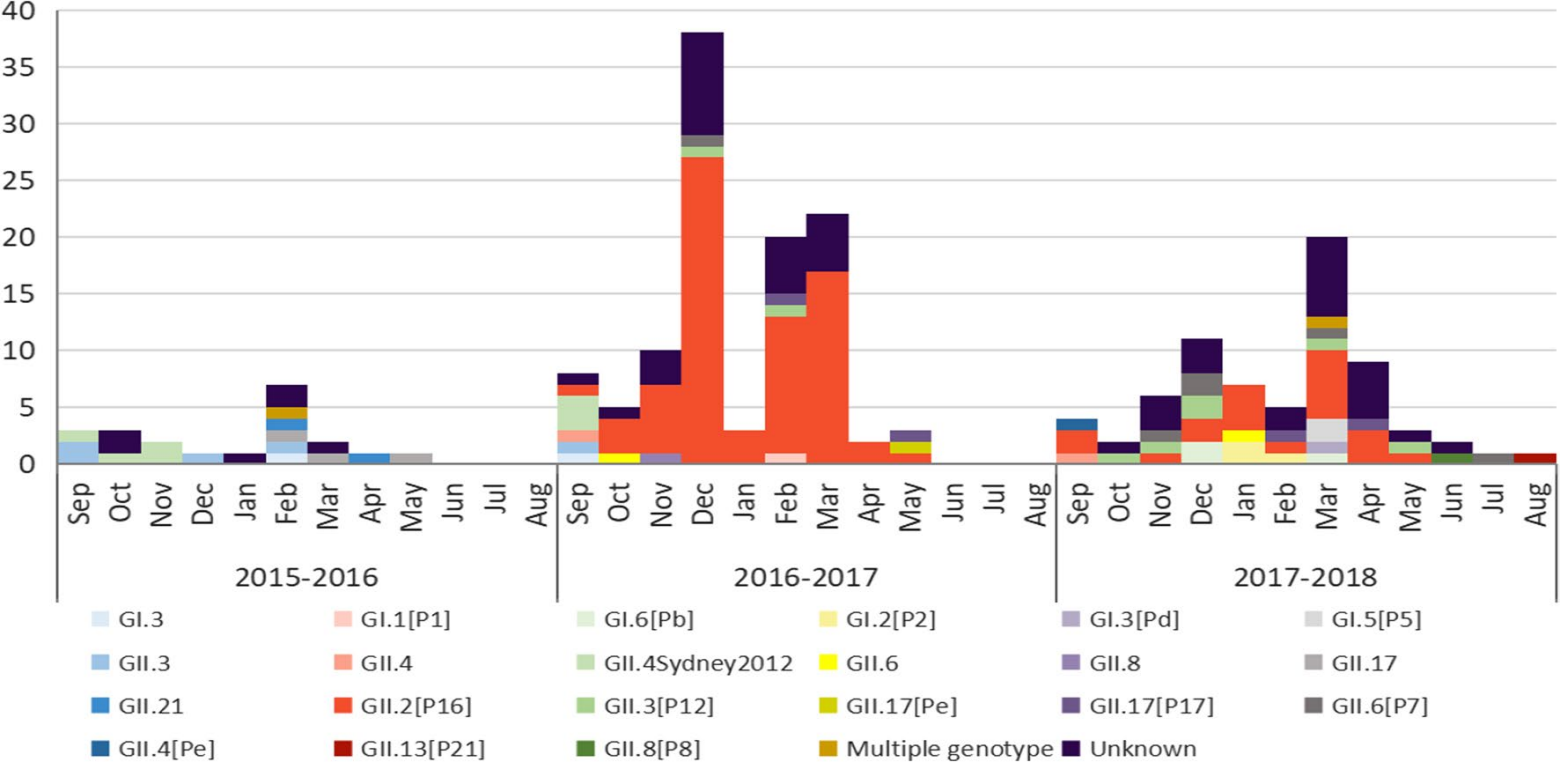
Monthly distribution of NoV outbreaks in Shenzhen by genotype. The numbers on the Y axis represent the number of NoV outbreaks

According to ten district-level CDCs, there were 203 NoV outbreaks in Shenzhen between the period September 2015 and August 2018. Most outbreaks were from Nanshan district (30.5%, 62/203) and no outbreak was from Yantian district (Fig. 1). Information on the outbreak size was reported for 197 (97.0%), ranging from 5 to 115 cases per outbreak (Table 1). Of the 203 outbreaks, 186 (91.6%, 186/203) occurred in school settings, with 17 (8.4%, 17/203) occurring in non-school settings (Table 2). Of the 186 outbreaks occurred in school settings, 143 (76.9%, 143/186) occurred in child care centres. The reported outbreaks peaked in the cold season, especially from November to March (Fig. 2).

**Table 1:** Number of people with NoV gastroenteritis per outbreak according to genotype

Genotype	N of ill people (range)	N of outbreaks involving ill people	Median no. of ill people (range)
GII.2	3–73	92	10
GII.3	3–45	13	7.5
GII.4Sydney 2012	9–14	7	11
GII.4	3–13	3	11
GII.6	4–8	8	6
GII.8	11–13	2	12
GII.17	3–12	8	5.5
GII.13	14	1	–
GII.21	11–23	2	17
Multiple genotype	3–10	2	6.5
GI.1	7	1	–
GI.2	5–9	3	7
GI.3	5–84	3	6
GI.5	3–5	2	4
GI.6	6–64	3	12
GII	3–115	53	9
Total	3–115	203	9

N denotes the number; ill people denotes symptomatic individuals

**Table 2:** Genotype distribution of NoV outbreaks from 2015 to 2018 according to setting

		No. (%) of outbreaks											
	Total	Child care centre	Primary school	Middle school	University	Hospital	Hotel	Restaurant	Work place	Nine-year school	Fifteen-year school	Institution	Community centre
GII.2	92 (45.3)	73 (79.3)	7 (7.6)	4 (4.3)	1 (1.1)	1 (1.1)	–	–	1 (1.1)	4 (4.3)	1 (1.1)	–	–
GII.3	13 (6.4)	11 (84.6)	1 (7.7)	–	–	–	–	–	–	1 (7.7)	–	–	–
GII.4Sydney 2012	7 (3.4)	6 (85.7)	1 (14.3)	–	–	–	–	–	–	–	–	–	–
GII.4	3 (1.5)	3 (100.0)	–	–	–	–	–	–	–	–	–	–	–
GII.6	8 (3.9)	5 (62.5)	2 (25.0)	–	–	–	–	–	1 (12.5)	–	–	–	–
GII.8	2 (1.0)	1 (50.0)	1 (50.0)	–	–	–	–	–	–	–	–	–	–
GII.17	8 (3.9)	2 (25.0)	1 (12.5)	–	–	–	1 (12.5)	1 (12.5)	2 (25.0)	–	–	1 (12.5)	–
GII.13	1 (0.5)	1 (100.0)	–	–	–	–	–	–	–	–	–	–	–
GII.21	2 (1.0)	–	–	–	–	–	1 (50.0)	–	–	–	–	1 (50.0)	–
Multiple genotype	2 (1.0)	–	–	–	1 (50.0)	–	1 (50.0)	–	–	–	–	–	–
GI.1	1 (0.5)	1 (100.0)	–	–	–	–	–	–	–	–	–	–	–
GI.2	3 (1.5)	1 (33.3)	1 (33.3)	–	–	–	–	–	–	1 (33.3)	–	–	–
GI.3	3 (1.5)	1 (0.7)	1 (4.3)	1 (14.3)	–	–	–	–	–	–	–	–	–
GI.5	2 (0.1)	2 (100.0)	–	–	–	–	–	–	–	–	–	–	–
GI.6	3 (1.5)	2 (66.7)	–	–	–	–	–	–	–	1 (33.3)	–	–	–
GII	53 (26.1)	34 (64.2)	8 (15.1)	2 (3.8)	2 (3.8)	1 (1.9)	1 (1.9)	1 (1.9)	1 (1.9)	1 (1.9)	–	1 (1.9)	1 (1.9)
Total	203 (100)	143 (70.4)	23 (11.3)	7 (3.4)	4 (2.0)	2 (1.0)	4 (2.0)	2 (1.0)	5 (2.5)	8 (3.9)	1 (0.5)	3 (1.5)	1 (0.5)

Nine-year school indicates schools that included primary school and junior high school; fifteen-year school indicates schools that included primary school, junior high school and senior high school; institution indicates facilities that belong to the government

### Genotypic distribution of identified NoV

Of the 203 outbreaks detected as caused by NoV according to real-time RT-PCR from September 2015 to August 2018, 150 were successfully genotyped. Of these 150 outbreaks with genotype information, 137 (91.3%, 137/150) and 12 (8.0%, 12/150) were classified into GII and GI genogroups. One (0.6%, 1/150) outbreak involved both GI- and GII-positive samples. The dominant genotype was GII.2[P16] (61.33%, 92/150). In addition, we identified a novel recombinant genotype GII.17[Pe] that had not been previously found in Shenzhen before (Table 3).

**Table 3:** Genotype distribution of identified NoV strains in Shenzhen, September 2015–August 2018

Genotype	2015.09–2016.08	2016.09–2017.08	2017.09–2018.08
	N (percentage)	N (percentage)	N (percentage)
*Capsid*			
GI.3	1 (4.8)	1 (0.9)	–
GII.3	4 (19.0)	1 (0.9)	–
GII.4	–	1 (0.9)	1 (1.4)
GII.4 Sydney2012	4 (19.0)	3 (2.7)	–
GII.6	–	1 (0.9)	1 (1.4)
GII.8	–	1 (0.9)	–
GII.17	3 (14.3)	–	–
GII.21	2 (9.5)	–	–
*RdRp/Capsid*			
GI.1[P1]	–	1 (0.9)	–
GI.6[Pb]	–	–	3 (4.3)
GI.2[P2]	–	–	3 (4.3)
GI.3[Pd]	–	–	1 (1.4)
GI.5[P5]	–	–	2 (2.8)
GII.2[P16]	–	73 (65.2)	19 (27.1)
GII.3[P12]	–	2 (1.7)	6 (8.6)
GII.17[Pe]	–	1 (0.9)	–
GII.17[P17]	–	2 (1.7)	2 (2.8)
GII.6[P7]	–	1 (0.9)	5 (7.1)
GII.4[Pe]	–	–	1 (1.4)
GII.8[P8]	–	–	1 (1.4)
GII.13[P21]	–	–	1 (1.4)
Multiple genotype	1 (4.8)	–	1 (1.4)
GII	6 (28.6)	24 (21.4)	23 (32.9)
Total	21 (100)	112 (100)	70 (100)

N denotes number

### Genotype distribution and outbreak characteristics

For outbreaks caused by the GII.2 strain, most occurred in school settings: 73 (79.3%, 73/92) outbreaks occurred in child care centres and the age distribution of GII.2 infection showed no significant differences (Fisher's exact test = 3.595, *p* = 0.177) through dominant school settings (child care centre, primary school, middle school). Of the thirteen outbreaks caused by the GII.3 strains, most (86%, 11/13) also occurred in child care centre.

### Phylogenetic analysis of the RdRp region and VP1 sequence of the GII.2[P16] strain

**Fig. 3:**
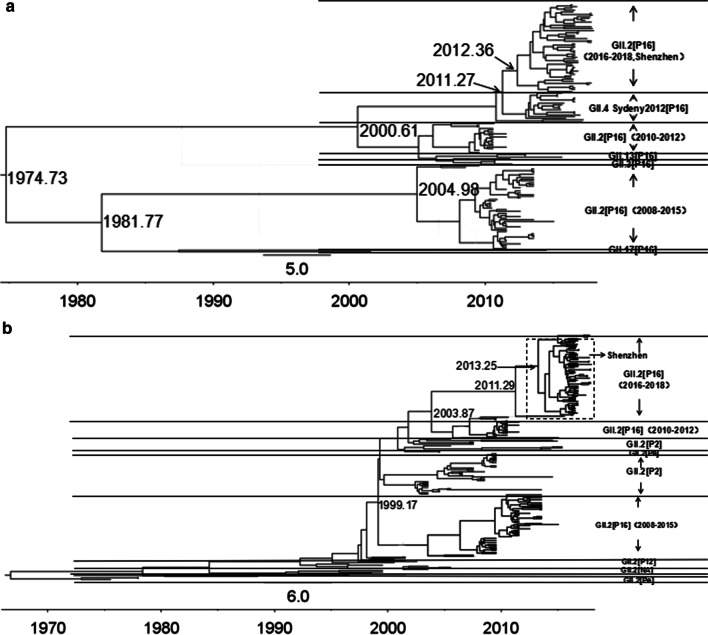
**A** Phylogenetic tree of the RdRp region of NoV GII.2[P16]. **B** Phylogenetic tree for the VP1 sequence of NoV GII.2[P16]. The scale bars denote the actual time (years). Estimated divergence times are shown on the ancestral nodes. Phylogenetic clusters, including the previous GII.2[P16] 2010–2012 cluster, the previous GII.2[P16] 2008–2015 cluster and the GII.P16/GII.2 2016–2018 cluster, are marked. The sequences of the GII.2[P16] 2016–2018 cluster were all collected from Shenzhen

To examine strain evolution, 52 full-length RdRp regions of strain GII.2[P16] sequences from Shenzhen and 95 reference sequences from GenBank were collected for analysis. According to the maximum clade credibility (MCC) tree, the evolutionary rate of the RdRp region of the GII.2[P16] strain was estimated as 2.1 × 10^–3^ (95% HPD interval, 1.7 × 10^–3^–2.5 × 10^–3^) substitutions/site/year. The common ancestors of the GII.2[P16] strain from Shenzhen and GII.4 Sydney 2012[P16] diverged from 2011 to 2012, and the RdRp region of the GII.2[P16] strain from Shenzhen showed genetic diversification during 2012 to 2013. The phylogenetic analyses suggested that the RdRp region clustered with GII.4 Sydney2012[P16] (Fig. 3A).

Simultaneously, 72 full-length VP1 sequences of GII.2[P16] retrieved from Shenzhen and 146 GenBank reference sequences were used to explore the evolutionary rate. The evolutionary rate of the VP1 sequence of the GII.2[P16] strain was estimated at 2.7 × 10^–3^ (95% HPD interval, 2.4 × 10^–3^–3.1 × 10^–3^) substitutions/site/year based on the MCC tree. The common ancestors of the GII.2[P16] strain from Shenzhen and previous GII.2[P16] (2010–2012) diverged from 2003 to 2004, and the VP1 sequence of the GII.2[P16] strain in Shenzhen showed genetic diversification from 2013 to 2014. The phylogenetic analyses suggested that VP1 clustered with GII.2[P16] (2010–2012) (Fig. 3B).

### Amino acid mutations of the non-structural region of GII.2[P16]

To explore the amino acid mutations within the non-structural region of the recombinant strains, 14 nearly full-length non-structural protein sequences and 22 full-length reference sequences, including GII.17[P16] (2002), GII.2[P16] (2009–2014), GII.2[P16] (2010–2012), GII.13[P16] (2015), GII.3[P16] (2012–2013), GII.4[P16] (2015–2016) and GII.17[P16] (2016–2018), from GenBank were aligned. Sequence data revealed 102 (6%) parsimony-informative sites, but no amino acid mutations in non-structural region of the GII.2[P16] recombinant strain. Furthermore, 6 amino acid substitutions (*77E, R750K, P845Q, H1310Y, K1546Q, T1549A) were found only in recent strains (GII.4 Sydney 2012[P16] and the GII.2[P16] recombinant strain), 2 sites (A644P, A1521V) were substituted in the GII.2[P16] recombinant strains, and 1 site (S/T753T) was reverted. The results showed that amino acid 1310 (motif G) was substituted (Table 4).

**Table 4:**
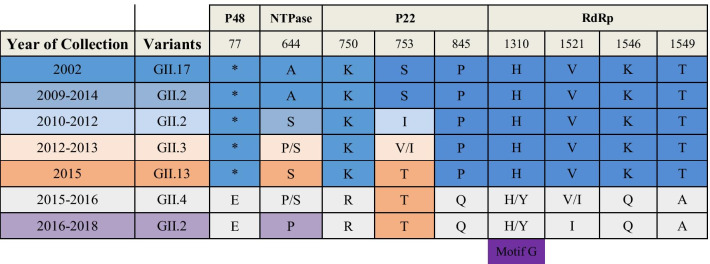
Amino acid mutation of non-structural region in NoV GII.P16

List of amino acid changes in the non-structural protein of the GII.2[P16] recombinant strain compared with those of previous GII.P16 NoV. Asterisks denote residue deletion in the P48 protein of GII.P16 noroviruses. The sequences of P48, NTPase, 3A protein, VPg, Pro and RdRp were aligned. Motif G is one of the conserved segments of RdRp according to function and structure, and the 1310 position belongs to Motif G. The negative results are not shown

### Amino acid mutations of HBGA-binding and epitope sites of the GII.2[P16]

To explore the HBGA-binding profile, predicted epitopes and epitope A to E sites of the GII.2[P16] recombinant strain [9, 23, 24], 72 full-length VP1 sequences from this study and 65 reference sequences, including GII.2[Pc] (1976–1978), GII.2[Ph] (1997), GII.2[P2] (1987–2015), GII.2[P12] (2004–2006), GII.2[P21] (2010), GII.2[Pe] (2014), GII.2[P16] (2010–2012), GII.2[P16] (2008–2014) and GII.2[P16] (2016–2018), from 1975 to 2018 were collected and aligned. Sequencing data revealed 29 parisimony-informative sites, but there were no mutations in the HBGA-binding profile, predicted epitopes and epitopes A to E of the GII.2[P16] strain (Additional file 1: Table S2).

## Discussion

In this study, NoV-associated AGE outbreaks in Shenzhen, China, from September 2015 to August 2018 were analysed. A total of 203 NoV outbreaks were reported to the Shenzhen CDC. NoV infection was initially described as "winter vomiting disease" due to its seasonal characteristics [25]. Analysis of the monthly distribution also indicated that the peak of the outbreak in Shenzhen occurred from November to March. Previous studies have found a link between climate or weather and increased NoV abundance, and low absolute humidity provides an ideal conditions for NoV persistence and transmission during cold months [26]. Indeed, NoV rapidly loses viability and infectivity with the increase in increasing temperature; therefore, NoV appears to be more stable in a cold climate and thus is transmitted more easily among people at cold times of the year [27, 28]. The peak in this study was in December, when Shenzhen began to become cold, and March, when the temperature began to turn warm, suggesting that that climate change has an impact on NoV transmission. The NoV outbreaks usually occur in hospitals, nursing homes, schools, childcare centres, hotels and other institutional settings [3]. A study in United States reported 3960 NoV outbreaks between 2009 and 2013 and found that long-term care homes were the most frequent sites of NoV outbreaks [29]. Another study from Qin et al. [30] showed that middle school was the most important setting of NoV outbreaks in China, followed by primary school between 2006 and 2016. In this study, we classified the outbreak settings into 12 categories, and the results showed that most were occurred in childcare centre, followed by primary school. This suggests that school remains the most common setting for NoV outbreaks in Shenzhen, but that the current high incidence is occurring among younger children who are under 6 years of age. Combining the results of the monthly distribution of NoV outbreaks in Shenzhen, we suspect that the decrease in the number of NoV outbreaks in January and February is related to school holidays. When the scale of the outbreaks was analysed, the average number of people involved per outbreak in Shenzhen was nine, smaller than the 18 persons reported in the United States [29]. Shenzhen is one of the cities where the economy is most developed, which may be a benefit of the local public health system and highly effective handling of public health emergencies in Shenzhen (http://www.szemo.gov.cn). Regarding genotype detection, both the GI and GII genogroups were found, as were 15 capsid types and 15 polymerase types. Among the genotypes, the most common was GII.2, followed by GII.3. GII.4 Sydney2012 only accounted for 3.4%. In this study, we identified the GII.2 strain as a GII.2[P16] recombinant strain similar to strains found in other regions in China [31, 32]. Moreover, the first outbreak identified as caused by the GII.2[P16] recombinant strain in Shenzhen was on September 30, 2016 after which the GII.2[P16] strain caused a steep rise in AGE in Shenzhen in the ensuing months. In general, recombination is thought to be important and common in virus evolution [33]. Most recombination occurs within ORF1/ORF2 overlapping regions or near the RdRp region, resulting in different capsid and RdRp genotypes [34]. In this study, we calculated the evolutionary rates of the RdRp region and VP1 sequence, which were 2.1 × 10^–3^ substitutions/site/year and 2.7 × 10^–3^ substitutions/site/year, respectively, indicating that the polymerase and capsid regions of NoV GII.2[P16] strains had evolved independently, which was consistent with the results of previous studies [15]. The evolution rate of NoV GII.2 was much lower than that of GII.4 NoV (4.4 × 10^−3^–7.4 × 10^−3^ substitutions/site/year) [35], suggesting that GII.2 was relatively stable in Shenzhen. Based on the evolutionary divergence time, the GII.2[P16] strains in Shenzhen might have recombined in 2013–2014 providing a better understanding of the formation of GII.2[P16] recombinant strains in Shenzhen.

The results of sequence alignment showed that important sites of VP1, including the HBGA-binding profile and epitope-predicted sites, were not mutated. This suggested that the reason for the prevalence of NoV GII.2[P16] strains in the population is different from that of the previous pandemic NoV GII.4, which was mainly due to changes in the capsid region leading to changes in blocking antibody epitopes to cause population among people [36, 37]. Parra et al. [37] analysed the GII.2 capsid sequences over a 40-year period and found only small differences, which agrees with our results, indicating that the GII.2 strain is more genetically stable than is the GII.4 strain. At the same time, the lack of variation in the antigen regions of strains may also explain their short duration. These results indicate that the presence of a structure other than the VP1 contributes significantly to the prevalence of the GII.2[P16] strain [38], which may help to reveal the reasons for the GII.17[P17] epidemic that caused the outbreak of acute gastroenteritis in many countries in the winter of 2014–2015. Tohma et al. [39] summarized the reasons for the epidemic caused by GII.17[P17] and believed it to be related to the non-structural region. In this study, amino acid substitutions were found within the nonstructural regions including P48, NTPase, P22 and RdRp. These non-structural proteins play important roles in NoV replication, damaging host cells and promoting virus synthesis by interfering with intracellular protein transport, vesicle misorientation and Golgi disintegration [40–42]. The RdRp region can be divided into three highly conserved segments according to function and structure, including the fingers, thumb, and palm subdomains, which can be organized into motifs A to G [43]. The results of amino acid mutation of non-structural protein sites of the GII.2[P16] recombinant strain suggest that the non-structural region may provide materials for virus replication, accelerate apoptosis in host cells and enhance fitness by changing the interaction mode. Another study also reported that the GII.2[P16] strain leads to a higher viral load than GII.4[Pe] and GII.17[P17] in patients [44]. However, not all changes in the non-structural region would cause epidemics. The study of Tohma et al. calculated the amino acid substitution sites in the RdRp region of GII.2[P2] and found that the replacement rate of GII.P2 was higher than that of GII.P16 [45]. Regardless, NoV GII.2[P2] outbreaks have not resulted in pandemics, indicating that the RdRp region plays a crucial role in the GII.2[P16] epidemic.

This study showed that the GII.2[P16] outbreaks have decreased in Shenzhen, although the continuous surveillance to monitor genotypes is still necessary to identify new variants in a timely manner. The limitations of this study were as follows. First, genotyping was only successful for 150 (73.9%) of the positive NoV cases in our study. Second, our study lacked clinical information and epidemiological data for outbreaks. In future studies, epidemiological surveillance should be more comprehensive and molecular analysis for different NoV genotypes should be developed.

## Conclusions

In conclusion, this study reported the epidemiological patterns and genetic characteristics of NoV in Shenzhen from September 2015 through August 2018, revealing that the main cause was the GII.2[P16] strain. This study also provides evidence that the evolution of the NoV GII.2[P16] strain occurred relatively slowly in Shenzhen.

## Supplementary Information

**Additional file 1: Table S1.** Primes used in this study. **Table S2.** Amino acid mutations of full-length VP1 sequences of the NoV GII.2. Reference sequences: Reference sequences used in this study.

## Data Availability

The datasets used in the current study are available from the corresponding author on reasonable request.

## Abbreviations

AGE: Acute gastroenteritis
NoV: Norovirus
RdRp: RNA-dependent RNA polymerase
HBGA: Histoblood group antigen
